# Deeper insight into speech characteristics of patients at ultra-high risk using classification and explainability models

**DOI:** 10.3389/fpsyt.2025.1595197

**Published:** 2025-06-16

**Authors:** Deok-Hee Kim-Dufor, Michel Walter, Marie-Odile Krebs, Yannis Haralambous, Philippe Lenca, Christophe Lemey

**Affiliations:** ^1^ Limics, Sorbonne Université, Université Sorbonne Paris-Nord, INSERM, Paris, France; ^2^ Unité de Recherche Clinique en Psychiatrie (URCP), Department of Psychiatry, Centre Hospitalier Universitaire (CHU) de Brest, Brest, France; ^3^ University of Paris, Groupe Hospitalier Universitaire de Paris (GHU)-Paris, Service Hospitalo-Universitaire, Sainte-Anne, Centre d'évaluation pour Jeunes Adultes et ADolescents (C’JAAD), Paris, France; ^4^ IMT Atlantique, Lab-STICC, UMR CNRS 6285, Brest, France; ^5^ Données, Modèles, Informations & Décisions (DECIDE), Department of LUSSI, Institut Mines-Télécom (IMT) Atlantique, Brest, France; ^6^ Consultation d’Evaluation de la VUlnérabilité Psychologique (CEVUP), Department of Psychiatry, CHU de Brest, Brest, France

**Keywords:** UHR patients, spoken language, natural language processing, XGBoost, SMOTE, SHAP values

## Abstract

**Introduction:**

Peculiar use of language and even language deficits are one of the well-known signs of schizophrenia. Different language features analyzed using natural language processing and machine learning have been reported to differentiate patients at ultra-high risk for psychosis. However, it has not always been explained how, and to what extent, those linguistic markers allow the distinction of patients. This study aims to find relevant linguistic markers for classifying patients at ultra-high risk and explain how the detected markers contribute to the classification.

**Methods:**

The first consultations with a psychiatrist of 68 patients (15 not-at-risk patients, 45 at-risk patients, and 8 patients with first episode psychosis) were recorded, transcribed verbatim, and annotated for analyses using natural language processing. A gradient-boosted decision tree algorithm was tested to evaluate its potential to correctly classify three categories of patients and find relevant linguistic markers at the level of lexical richness, semantic coherence, speech disfluency, and syntactic complexity. The Synthetic Minority Oversampling Technique was used to handle imbalanced data, and the SHapley Additive exPlanations (SHAP) values were computed to measure feature importance and each feature’s contributions to the classification.

**Results:**

The model yielded good performance, that is, 0.82 accuracy, 0.82 F2-score, 0.85 precision, 0.82 recall, and 0.86 ROC–AUC score, with four linguistic variables that concern weak coherence, the use of “I,” and filled pauses.

**Discussion:**

The findings in this study suggest that weak coherence play a key role in classification. No significant differences in the use of “I” and filled pauses were found between groups using a statistical test, but an explainability model showed its different contributions. The contribution of each linguistic feature to the classification by patient group provided deeper insight into linguistic manifestations of each patient group and their subtle differences, which could help better analyze and understand patients’ language behaviors.

## 1 Introduction

People with schizophrenia present with significant impairments stemming from disordered cognitive functioning (1). This mental illness manifests itself in characteristic symptoms such as delusions, hallucinations, disorganized thinking and behaviors, limited speech and expression of emotions, and social withdrawal. Early detection and treatment of schizophrenia have been proven to lead patients to favorable prognosis and better quality of life (2, 3). They could indeed reduce the risks and disorders associated with the first symptoms by engaging patients who present with prodromal symptoms in a care pathway (4) and limit the duration of untreated psychosis (DUP) by means of a treatment at the onset of the first episode of psychosis (FEP). The DUP is one of the key prognostic factors both in FEP (5) and in chronic schizophrenia (6). Different clinical assessments allow prodromal symptoms to be identified such as the Comprehensive Assessment of At-Risk Mental States (CAARMS), the Structured Interview of Psychosis-risk Syndromes (SIPS) from the “Ultra-High Risk (UHR)” criteria, and the Schizophrenia Proneness Instrument—Adult (SPI-A) from the basic symptom concept. Even though these tools show acceptable or fairly good performances, they still have a somewhat limited rate of prediction (7). Complementary elements for better predictions have therefore become a desideratum, and natural language processing (NLP) comes into play. Peculiar uses of language in schizophrenia (8–10) have been reported in the literature and are one of the well-known signs (11, 12). They are very easily noticeable and even qualified as “schizophrenic language” and “schizophrenese” by some authors in the last century (13–16). Peculiarities are observed at different language levels ranging from words to sentence structure, coherence, pragmatics (17–21) as itemized in the Scale for the Assessment of Thought, Language, and Communication by Andreasen like neologism, word approximation, poverty of speech, poverty of content, tangentiality, derailment, incoherence, and stilted speech (8). Based on the idea that self-disturbance is one of the core features of schizophrenia, a phenomenological approach to the sense of self in patients has developed (22–24) along with studies on the use of first-person pronouns (25–29). Language analysis of syntactic variables was already proposed in the 1980s as a potential diagnostic aid (30–32), since differences were observed between schizophrenics, maniacs, and controls (30, 31). Even though language analyses turned out to have great potential, they were highly time consuming and likely to be subjective because they had to be manually carried out. Automated language analyses are more objective methods and unlimited in data size. Many studies have therefore explored language in schizophrenia and searched for linguistic markers to be used as a diagnostic aid along with biomarkers such as brain imaging, genetic testing, and blood tests (33–35). With the development of artificial intelligence, analysis techniques, such as NLP and machine learning (ML) models, have become more sophisticated and yielded more propitious results. These techniques have been used on linguistic data in a growing number of studies on mental health (36, 37), namely, those on schizophrenia and FEP (38, 39): latent semantic analysis for quantifying speech coherence (40), semantic, lexical, and pragmatic features (41–44), speech graph connectivity for measuring thought disorder in schizophrenia and mania (45, 46) and for predicting transition (47, 48), longitudinal classification of FEP (49), clustering for constructing language profiles of heterogeneous linguistic behaviors of patients with schizophrenia for early intervention (50) and prognosis (51), and a combination of acoustic and semantic features for classifying schizophrenia-spectrum disorders (52), to name a few. The aims of this exploratory study were to detect relevant language features that could classify patients by their status at their first consultation with a psychiatrist and seek to explain classification results with respect to clinical observations. Among the linguistic markers found in these studies (40–51), the most frequent language feature is semantic coherence despite different types and lengths of corpus. It was therefore hypothesized that semantic coherence would be part of the relevant linguistic markers in conversational discourses of patients at ultra-high risk. With the disturbed sense of self observed in the clinic, it was also hypothesized that the use of first-person singular pronoun would vary depending on the UHR patient groups.

## 2 Methods

### 2.1 Participants

Sixty-eight patients (34 males, 34 females; mean age = 19.3 ± 2.86) participated in the present study. Out of the 68 patients, 15 were assessed as NAR (7 males, 8 females; mean age = 19.5 ± 2.24), 45 as AR (22 males, 23 females; mean age = 19.2 ± 2.83), and 8 as FEP (5 males, 3 females; mean age = 19.7 ± 3.78) using the CAARMS at T0. In total, 33 patients had antidepressants and/or anxiolytics, 5 were under neuroleptic treatment for less than 6 months, and 20 had no drug treatment. Healthy controls were not recruited separately to respect the same conditions of collecting data for each of the three groups, that is, a consultation with a psychiatrist. All were native speakers of French with an IQ superior to 70 and were informed of the study. Education levels were as follows: NAR [years of education (YoE) = 12.07 ± 1.34], AR (YoE = 11.58 ± 1.32), and FEP (YoE = 12 ± 1.73). A statement of non-opposition to the study was signed by their physician or the parents of underage patients.

### 2.2 Collection of patients’ speech and transcription

The recruited patients were recorded during their first consultations with a psychiatrist at the Center for Evaluation of Psychological Vulnerability (CEVUP) of the University Hospital of Brest, France. The first consultation with a psychiatrist is the starting point of the care pathway at the CEVUP. It is therefore labeled T0 (time zero), and a 2-year follow-up is indicated as T2. The interviews are semi-structured with some predetermined questions on the patient’s problems. The topics broached are the patient’s background, family, social relationships, socio-professional insertion, complaints about their symptoms, and any other topics based on what is said by the patient. Some additional questions are asked if more detailed information is needed for better understanding of the help seeker’s problems to assess their risk for psychosis. The transcripts have a conversational form between a psychiatrist and a patient. A nurse participated in the consultations, but she seldom spoke, and even when she did, it was only to provide the patient with supplementary information on the care pathway at the end of the consultations. The total duration of each recording is approximately 1 h. The mean total number of all words is 4,979.18 (SD = 2,448.70). The entire utterances including filled pauses, neologisms, and mispronunciations were transcribed verbatim using Microsoft Word by two trained assistants with clear instructions. Each speech turn starts on a new line and that of the healthcare provider is marked with an octothorpe (#) at the beginning and at the end. The present study has been approved by the IRB—Comité de Protection des Personnes EST-III (CPP:18.04.03, ID-RCB: 2017-A02702-51).

### 2.3 Preprocessing

An experienced linguist carried out preprocessing following predefined instructions. The spellings were manually double checked and corrected in all the transcripts without affecting their verbatim nature. Three different symbols, inspired by the method proposed by Foster and colleagues (53), were used to mark the elements required for analyses as follows:

- **{}** for speech disfluency such as filled pause, repetition, false start, auto-correction, and auto-interruption/abandonment
- **|** for clauses whose nucleus is a conjugated verb
- **< >** for minor utterances (no conjugated verbs).

The transcripts were segmented in three ways: each speech turn as a segment, each sentence as a segment, and each sentence without the healthcare provider’s speech as a segment. For the first segment, each new line was a segment; for the second, each punctuation; and for the last, the whole new lines starting and ending with octothorpes were removed using Python as well as the blank lines generated by this removal process.

### 2.4 Linguistic variables

The preprocessed transcripts were analyzed using NLP techniques with Python, which resulted in 33 features at the lexical, syntactic, and semantic levels and that of speech fluency (see Table in **Supplementary Material**).

#### 2.4.1 Lexical level

Lexical richness was measured to explore the variety of words and the quality of vocabulary. For the former, lexical diversity was calculated using the type–token ratio (54). For the latter, the proportion of content words (nouns, verbs, adjectives, and adverbs) to the total number of words, called lexical density (55), was measured. Since function words are excluded, lexical density reflects how informative the discourse is. Disturbed self-experience and different patterns of use of the first-person singular pronoun in people with schizophrenia have been reported (26, 29, 56). The use of personal pronouns was explored through three different measures as follows: the proportion of “I” to the total number of subject personal pronouns, the proportion of “I” to the total number of words, and the ratio of the first-person singular subject pronoun to the first-person object pronoun. The analyses at the lexical level were carried out on the lemmatized corpus using treetaggerwrapper (57).

#### 2.4.2 Syntactic level

Syntactic complexity and poverty of speech were measured. The analyses were based on lexicogrammatical constituency in functional grammar. Constituency is the hierarchical compositional structure of language, and this hierarchy of units is denominated as a rank scale, with each step in the hierarchy referred to as one rank (58). The ranks of lexicogrammatical constituency are clause > phrase/group > word > morpheme, wherein the clause is the highest unit and the central processing unit. In addition, this unit is one of the five levels in the grammatical system (59) and the primary unit in immediate speech processing (60). The clause has therefore been determined as the basic syntactic unit in this study. The utterances were segmented into clauses whose nucleus is a conjugated verb. When a group of words lacks a conjugated verb, it is considered a minor utterance. As for syntactic complexity, Szmerecsány compared syntax tree-based node counts, length-based word counts, and index of syntactic complexity calculated based on subordinators and embeddedness with regard to their accuracy and applicability (61). The results showed that all the three methods were almost perfect proxies, and therefore the most economical method, word counts, could be used. The average number of words per clause was therefore calculated as a measure of syntactic complexity. In turn-taking between a patient and a psychiatrist, the number of the patient’s turns was counted, and the proportion of the turns only with minor utterances (short answers) to the total number of their turns was calculated. A patient’s turn is considered minor utterance when the patient answers with simple words such as “yes,” “no,” “OK,” or a group of words without developing the reply. For example, to the question “How are you feeling today?”, the reply would be “so so/a little better/not really happy about all this.” This type of utterances is in line with “poverty of speech,” which is widely described in the literature (8, 10, 12). All the disfluency elements have been removed from the corpus prior to the syntactic analyses.

#### 2.4.3 Semantic level

Latent semantic analysis (LSA) (62, 63) has been applied to measure incoherence in speech (40, 41) and turned out to be fairly efficient when combined with other linguistic features (41–43, 49). LSA is a widely used NLP technique that analyzes texts to explore the relationships between a set of documents and the terms inside those documents. The underlying idea of LSA is that semantically similar words occur in similar texts, and thereby the cooccurrences of terms in large corpora of texts are used for measuring the lexical proximity/semantic similarity of terms of a language. LSA was chosen over other techniques for the following assets: a) the technique is based on a psychological theory of meaning and has shown results similar to human evaluations in educational applications (63); b) early studies using this technique paved the way for the use of NLP in early detection of psychosis (40, 41, 64, 65); c) LSA can handle longer passages of words (66) and synonyms in case of word redundancy for the avoidance of repetition (63); and d) contrary to new transformer-based models, this technique is not sensitive to initialization parameters, which allows consistent results. In addition, an LSA-based text analysis tool called Coh-Metrix (67, 68) has been efficiently used in studies on formal thought disorder (FTD) (56, 69–71). In the present study, semantic coherence was measured in three different types: intersubjective, subjective, and subjective without doctor (abbreviated henceforth as *wodr*) coherence. In the first type, semantic coherence was measured based on turn-taking, which represents dialogue coherence, inter-turn comparison; in the second, based on punctuation marks, such as periods and question marks, which could be called sentence-to-sentence coherence; and in the third, only the patients’ speech was considered. For the semantic analyses, the transcripts were not lemmatized (72), stop words were removed, and the disfluency elements were kept for the sake of semantic integrity.

#### 2.4.4 Speech fluency

Speech flow can vary in any individuals depending on their situation, state of mind, and/or fatigue. Disfluencies in speech comprise unfilled pauses (silent), filled pauses (“*uh*,” “*um*”), false starts, repetitions, autocorrection, parenthetical remarks (“*well*,” “*yeah*”) (73), and abandoned utterances (abandonment/auto-interruption). Various features of speech disfluency in patients with psychotic disorders, such as filled pauses, autocorrection, reparandum–interregnum repair structure, and unfilled pauses, have been studied in detail (74–76). All the disfluency elements, except unfilled pauses, were counted, and three disfluency-related subcategories were created as features in the present study as follows: filled pauses, abandonments/auto-interruptions, and auto-corrections/repetitions/false starts. The proportion of each of the three to the total number of words was calculated. A disfluency element with several words was counted as one. Among the abandoned utterances, clauses with a subject and an incomplete predicate have constituted a variable, that is, truncated clauses.

### 2.5 Statistics, XGBoost Classifier, SMOTE, SHAP values

Statistical analyses were carried out using Python scipy (77) and statsmodels (78). Data normality was tested using Kolmogorov–Smirnov test. For group comparisons in each of the 33 linguistic features and education levels, a Kruskal–Wallis test and a Dunn–Bonferroni test, as a *post hoc* analysis, were performed. Data homoscedasticity was verified using Levene’s test. A Kendall’s tau-b was calculated between the linguistic variables and the patients’ education levels as possible confounders.

**Figure 1:**
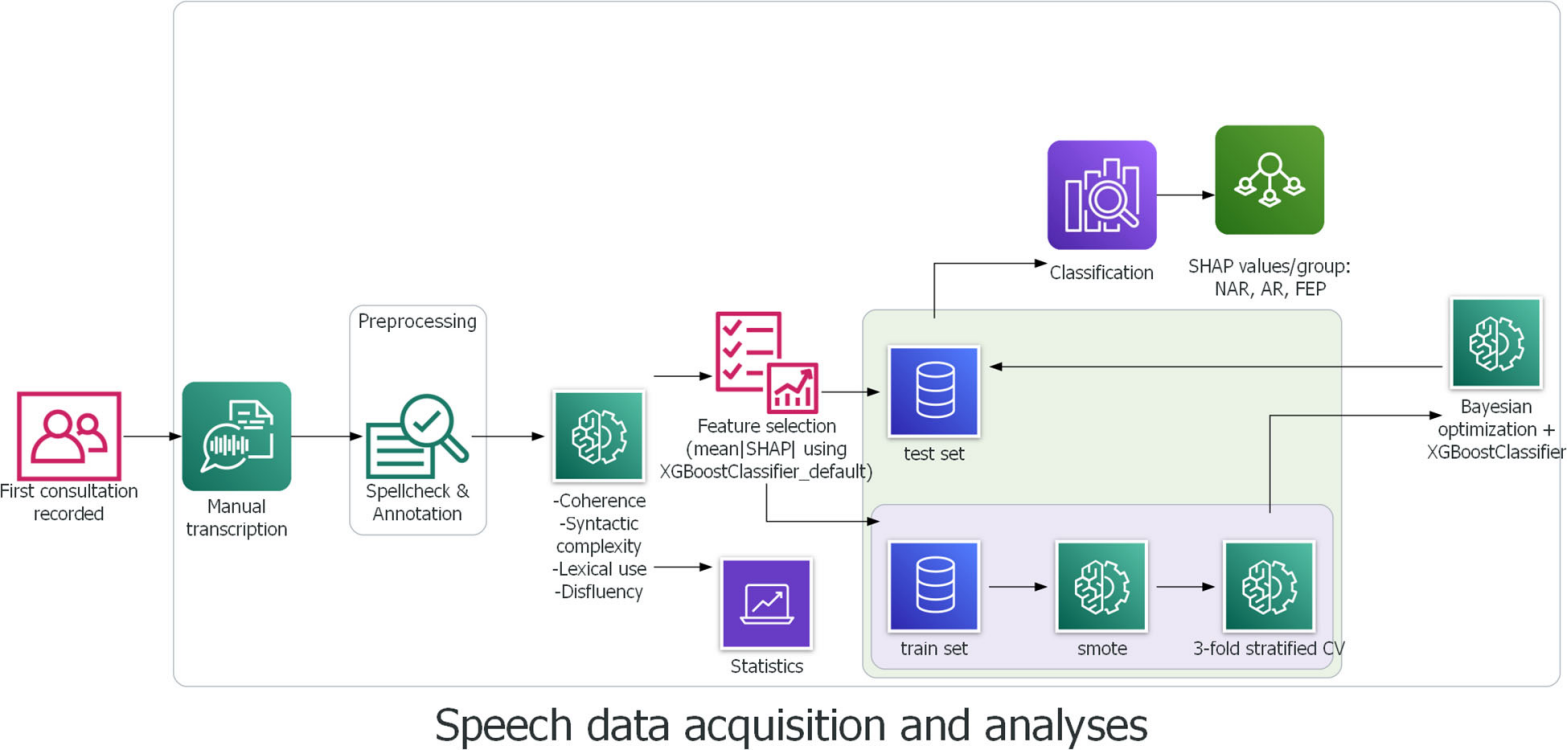
Pipeline for speech data acquisition and data analyses.

A supervised machine learning model XGBoost, for eXtreme Gradient Boosting (79) was used for classification. The gradient boosting method provides higher predictive accuracy thanks to its functional characteristics, that is, it combines weak learners to give rise to a stronger learner and therefore forms a more robust model (80). In addition, multicollinearity does not affect the stability and robustness of the model’s performance thanks to the capability of the algorithm to choose the best of highly correlated features (81). Furthermore, XGBoost has shown better performance with small datasets (82, 83) than other classifiers. The dataset in the present study is imbalanced. This limitation was addressed through SMOTE (Synthetic Minority Oversampling Technique) (84), a statistical technique for upsampling the minority class for a better balanced dataset. This technique has already been used and proven its efficacity, for example, in diagnosis, classification, and prognosis of cancer, diabetes, and Parkinson’s disease (85–97) to name a few. Stratified K-fold cross validation (k = 3) was used to split the data into train and test sets, and SMOTE was subsequently conducted individually in each fold to avoid data leakage. Stratified K-fold cross validation was chosen over leave-one-out cross validation for the sake of computational time and power, and k = 3 was set considering our relatively small dataset and the number of patient groups. The test size was 0.3. Using Bayesian Optimization (98) to tune hyperparameters, an XGBoost Classifier was trained using the 33 features of the original data to compute the SHapley Additive exPlanation (SHAP) values (99), and the mean absolute SHAP values were calculated for feature selection (100, 101). Another XGBoostClassifier was then trained using the outcome of feature importance based on the mean absolute SHAP values and the upsampled data. Inspired by Shapely values (102) from cooperative game theory, the SHAP values allow interpreting the model output by measuring the contribution of each feature to predictions. Precisely, the SHAP values reveal how much (magnitude) and either positively or negatively (direction) each feature affected the classification (99). This method thereby allows explanations and better interpretation of the results. The process of speech data acquisition and analyses is depicted below in **Figure 1**.

## 3 Results

### 3.1 Statistical results

A Kolmogorov–Smirnov test showed that no feature had a normal distribution (0.5 ≤ *D* ≤ 1 and p < 0.00 in all 33 features). The results of Levene’s test indicated homogeneity of variance in all features (p > 0.05). A Kendall’s tau-b test showed no evidence for a moderate or strong impact of years of education on the linguistic features (*r_τ_* = 0.24, p = 0.01 between average number of words per clause and education level; −0.14 ≤ *r_τ_* ≤ 0.16, 0.07 ≤ p ≤ 0.99 in all the other pairs). A Kruskal–Wallis test was performed on each of the 33 features of the three groups. The results revealed significant differences between the three groups in two features (intersubjective LSA minimum and subjective LSA minimum) as shown in **Table 1a** (for the full table, see **Supplementary Material**). A Dunn–Bonferroni test was then conducted to verify which groups were different. Its results indicated significant differences either between AR and FEP or between AR and FEP, but no differences were found between NAR and AR as shown in **Table 1b**.

**Table 1:** Kruskal–Wallis test results of the main features (a) and Dunn–Bonferroni test results (b).

(a)						
Features	Total	df	H	Effect size (ϵ2)		p-Value
Intersubjective LSA median	68	2	2.4011	0.0358		0.3010
**Intersubjective LSA minimum**	**68**	**2**	**13.4282**	**0.2004**		**0.0012**
Subjective LSA median	68	2	2.1901	0.0327		0.3345
**Subjective LSA minimum**	**68**	**2**	**8.2831**	**0.1236**		**0.0159**
Subjective LSA wodr median	68	2	2.3885	0.0356		0.3029
Subjective LSA wodr minimum	68	2	1.9917	0.0297		0.3694
Intersubjective LSA IQR	68	2	3.3373	0.0498		0.1885
Intersubjective LSA +1.5IQR %	68	2	2.7397	0.0409		0.2541
Intersubjective LSA −1.5IQR %	68	2	4.6137	0.0689		0.0996
Subjective LSA IQR	68	2	3.3373	0.0498		0.1885
Subjective LSA +1.5IQR %	68	2	2.7397	0.0409		0.2541
Subjective LSA −1.5IQR %	68	2	4.6137	0.0689		0.0996
Subjective LSA wodr IQR	68	2	3.3373	0.0498		0.1885
Subjective LSA wodr +1.5IQR %	68	2	2.7397	0.0409		0.2541
Subjective LSA wodr −1.5IQR %	68	2	4.6137	0.0689		0.0996
Lexical diversity (%)	68	2	2.7213	0.0406		0.2565
je (%)_total n	68	2	5.3242	0.0795		0.0698
je (%)_pp	68	2	2.4731	0.0369		0.2904
Ure's lexical density (%)	68	2	3.1329	0.0468		0.2088
Truncated clauses (%)	68	2	0.5767	0.0086		0.7495
Short answers (%)	68	2	2.3239	0.0347		0.3129
Ratio_subj/obj	68	2	1.5512	0.0232		0.4604
Filled pauses	68	2	4.4079	0.0658		0.1104
Abandonment	68	2	0.8165	0.0122		0.6648
Autocorrection_Repetition	68	2	0.5039	0.0075		0.7773
(b)						
Features	NAR vs. AR (*p*)		NAR vs. FEP (*p*)		AR vs. FEP (*p*)	
Intersubjective LSA minimum	0.1336		**0.0007**		**0.0267**	
Subjective LSA minimum	1.000		0.0696		**0.0123**	

The rows in bold are features and values with a significant difference (*p* < 0.05).

### 3.2 Classification and explainability results

**Figure 2:**
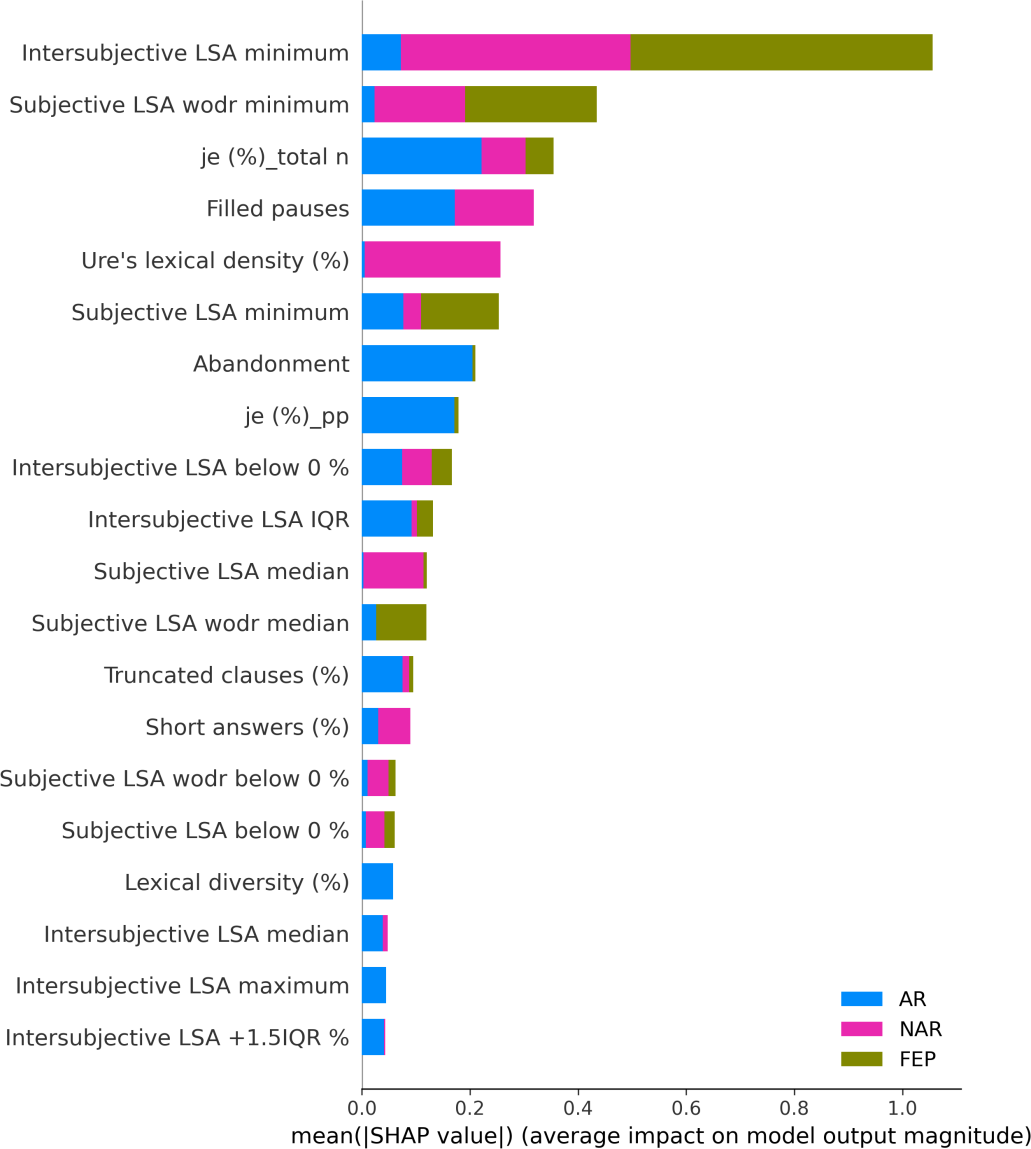
Mean absolute SHAP values.

**Figure 3:**
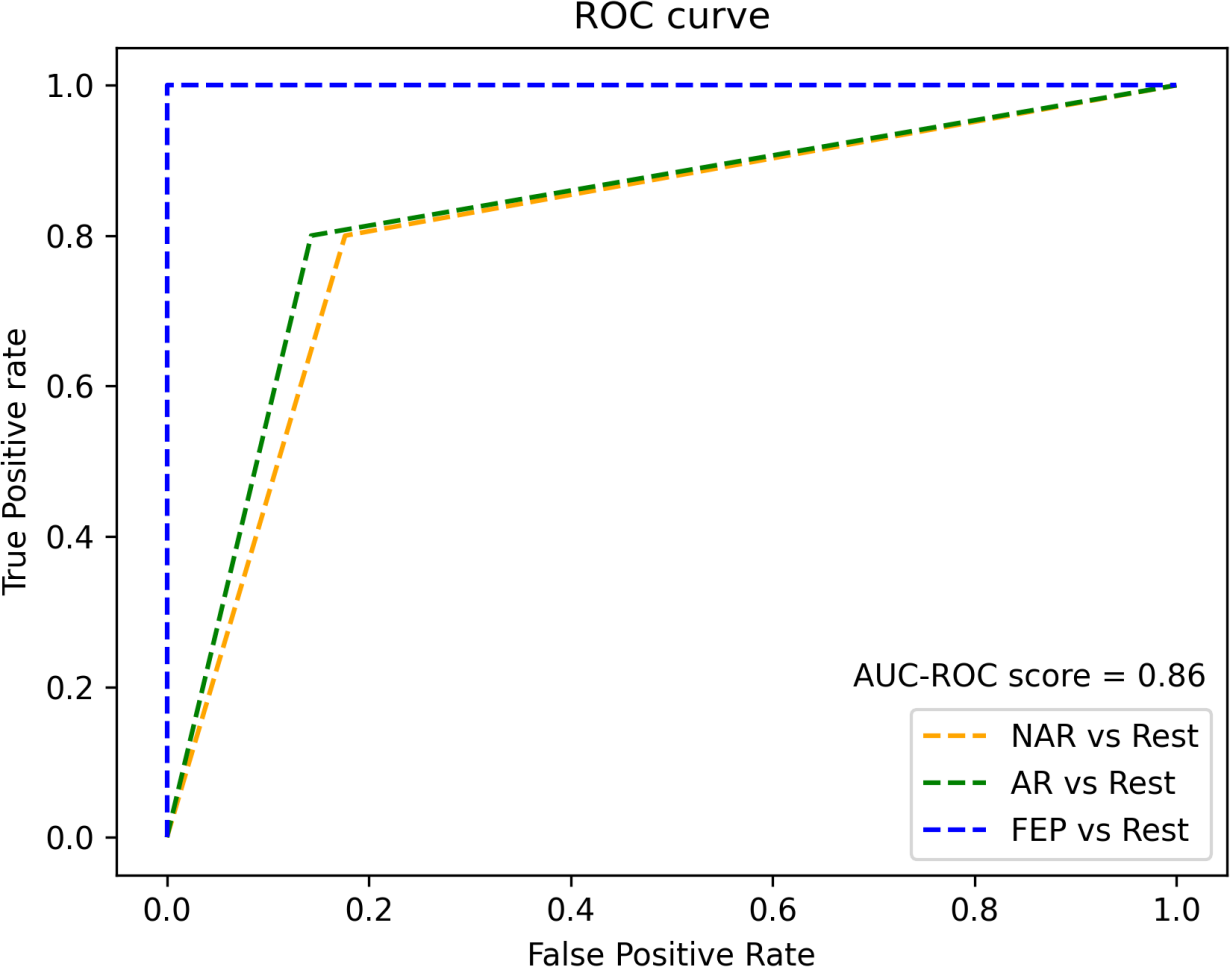
ROC curve of XGBoostClassifier model.

The XGBoostClassifier trained on SMOTE data with all the features yielded 0.75 accuracy, 0.73 precision, 0.75 recall, 0.74 F2-score, and 0.70 ROC–AUC score. The most impactful features were selected based on the mean absolute values computed on the original data as shown in **Figure 2**. The first four features whose values are greater than 0.3 were selected (intersubjective LSA minimum, subjective LSA wodr minimum, the proportion of “I” to the total number of words, and filled pauses) for another classification using XGBoostClassifier. This cutoff selection was based on threshold tests on the first 10 features. The best result was obtained when the first four features were included; for example, with the first five features, the accuracy was slightly lower (0.79) than that with the first four features and higher than that with the whole features (0.75). The newly trained model reached 0.82 accuracy, 0.85 precision, 0.82 recall, 0.82 F2-score, and 0.86 ROC–AUC score (see **Figure 3** for ROC–AUC curve), and as for 95% confidence intervals (CI) of accuracy, the lower CI was 0.68 and the upper CI, 0.95. The specificity and sensitivity of each group (group-specificity–sensitivity) were as follows: NAR-0.82–0.80, AR-0.86–0.80, and FEP-1.00–1.00. The results are shown in **Table 2**. Eight patients in the test set had their statuses at T2. Only one AR patient at T0 was misclassified into NAR by our model, but their status at T2 turned out to be NAR.

**Table 2:** Classification report (a), specificity and sensitivity (b), 95% confidence intervals (c).

(a) Classification report				
Patient group and Metrics	Precision	Recall	F1-score	Support
NAR	0.57	0.80	0.67	5
AR	0.92	0.80	0.86	15
FEP	1.00	1.00	1.00	2
Accuracy			0.82	
Macro average	0.83	0.87	0.84	
Weighted average	0.85	0.82	0.83	

(b) Specificity and sensitivity by group				
Patient group	Specificity		Sensitivity	
NAR	0.82		0.80	
AR	0.86		0.80	
FEP	1.00		1.00	

(c) 95% CI				
Accuracy and Confidence level	95% Confidence interval (CI)			
Test accuracy	0.82			
Lower CI	0.68			
Upper CI	0.95			

**Figure 4:**
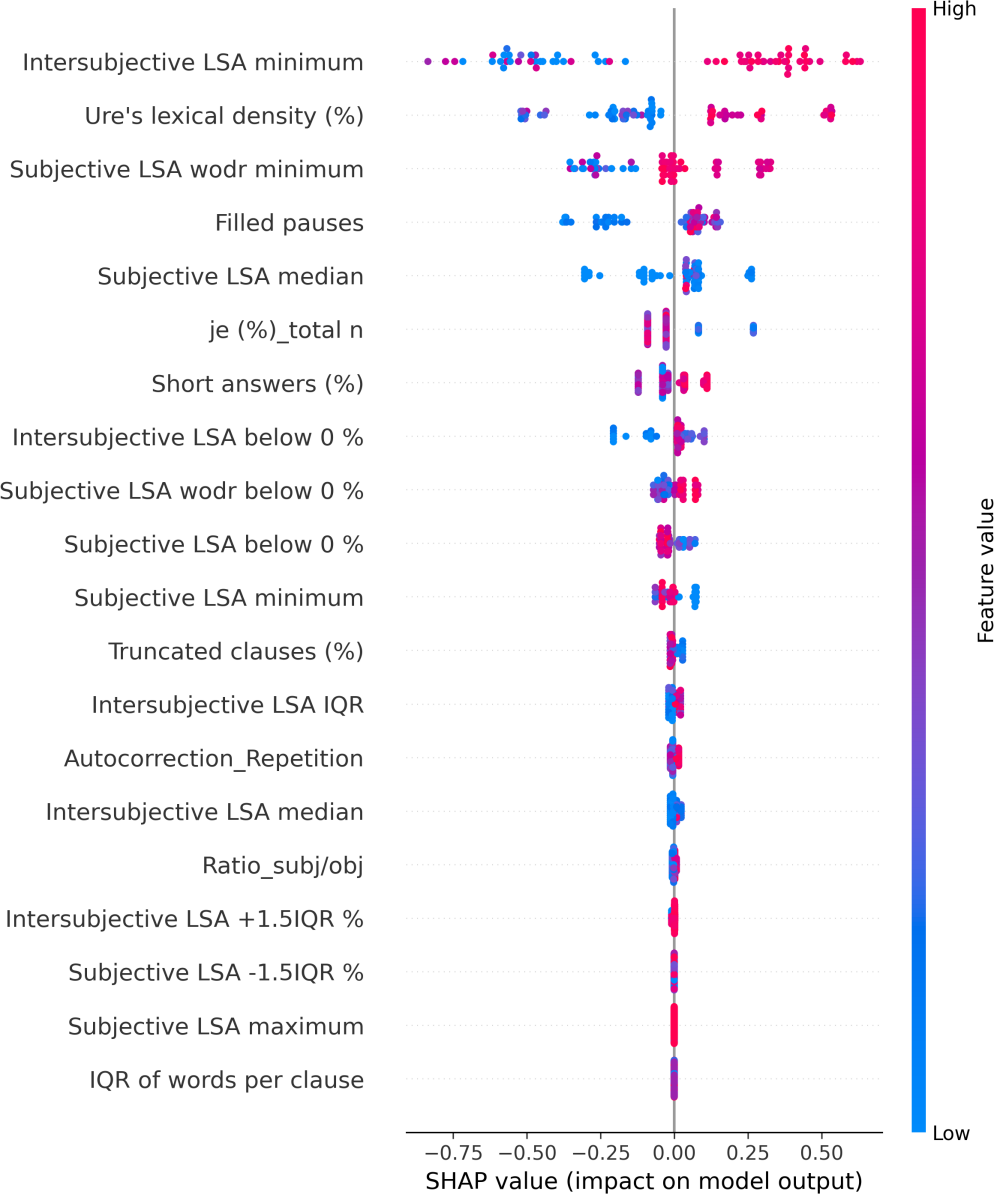
SHAP values of Not-At-Risk patients.

The SHAP values of each individual in each class are visually represented in **Figures 4** (NAR), **5** (AR), and **6** (FEP). The x-axis indicates the SHAP values, the y-axis shows the features, and the color of the point represents the original value of that sample, that is, higher in red and lower in blue. The farther a point is from the center vertical axis, the stronger its impact is on the classification. **Figure 2** shows that lower scores in intersubjective LSA minimum, lexical density, and subjective LSA without doctor minimum have a negative impact on predictions. In other words, these lower values are indicative of the individuals’ lower chance of being classified as NAR. Conversely, higher scores, albeit to a lesser degree, in filled pauses and subjective LSA median contribute positively to NAR. The magnitude of the higher scores in the proportion of “I” to the total number of words suggests their relatively small negative impact on the NAR classification. In **Figure 3**, the lower proportion of “I” to the total number of words, and higher frequencies of abandonment/auto-interruption and filled pauses, have a negative impact on predictions in AR. When scores in the proportion of “I” to the personal pronouns and subjective LSA minimum are higher, the odds on individuals being classified as AR are higher. **Figure 4** shows that lower minimum scores in all the three types of LSA contribute positively to FEP with the greatest magnitude of intersubjective LSA minimum. Higher values in subjective LSA wodr median negatively impact FEP. The contributions are summarized by patient group, direction, and magnitude in **Table 3**.

**Figure 5:**
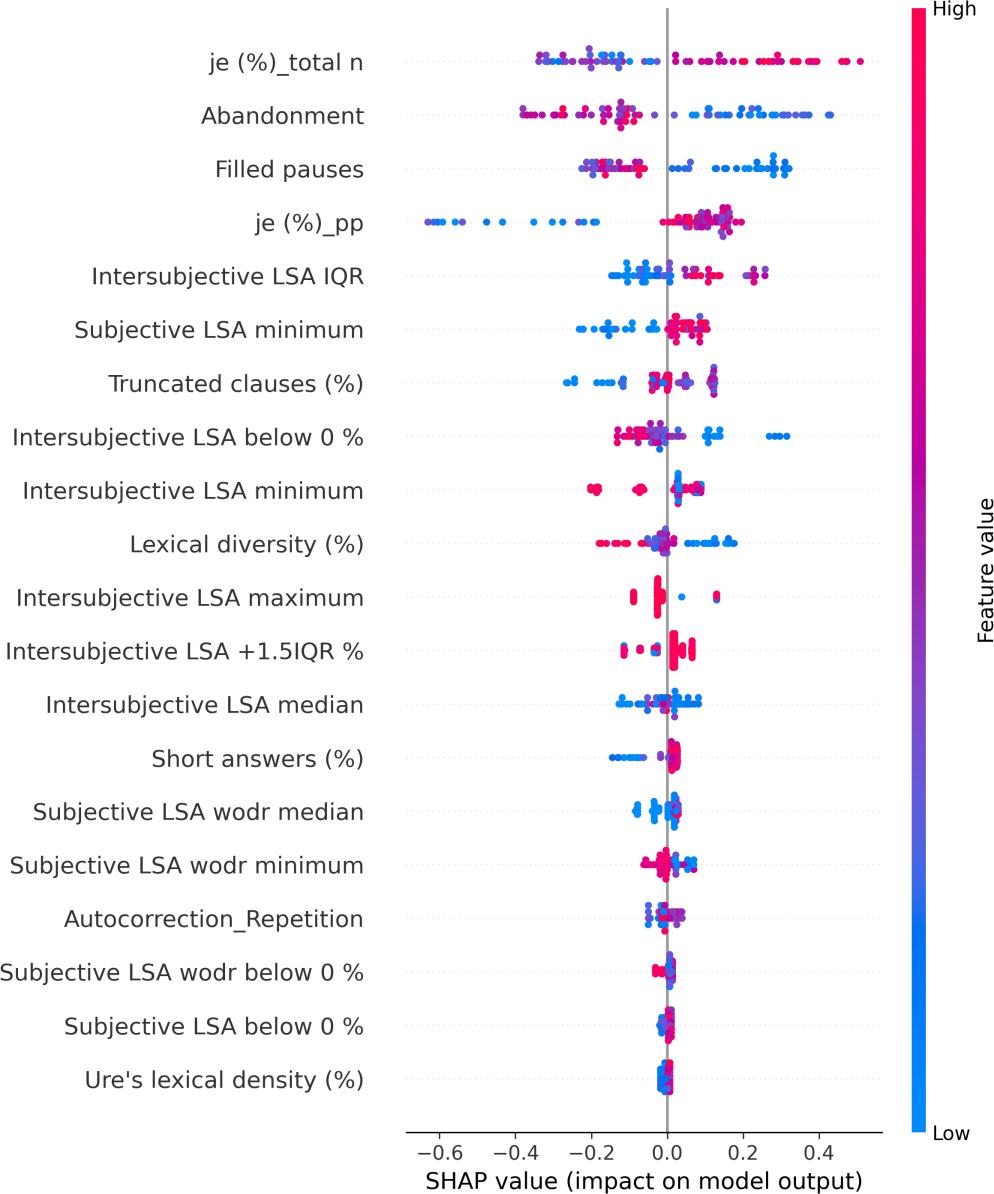
SHAP values of At-Risk patients.

**Figure 6:**
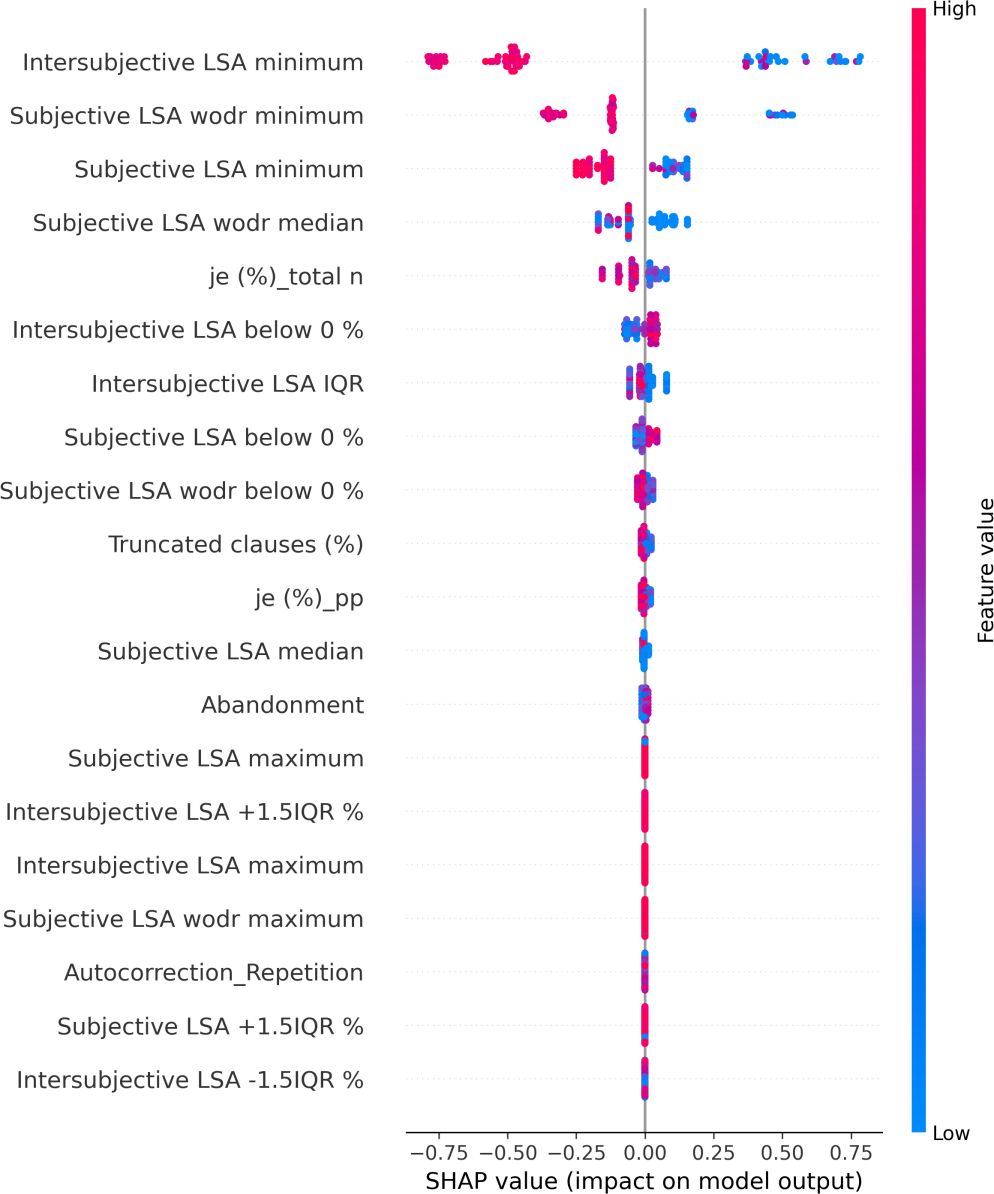
SHAP values of First Episode of Psychosis patients.

**Table 3:** Overview of the directions (positive and negative impacts on classification) and magnitudes (higher and lower values marked with ordinal numbers) of linguistic markers based on SHAP values.

Patient groups	Positive impact on classification		Negative impact on classification	
	Higher values	Lower values	Higher values	Lower values
NAR	Filled pauses (4th)		“I”/total (5th)	*Intersubjective LSA minimum* (1st)
	Subjective LSA median (6th)			Lexical density (2nd)
				Subjective LSA wodr minimum (3rd)
AR	“I”/personal pronouns (4th)		Abandonment/auto-interruption (2nd)	“I”/total (1st)
	*Subjective LSA minimum* (5th)		Filled pauses (3rd)	
FEP		*Intersubjective LSA minimum* (1st)	Subjective LSA wodr median (4th)	
		Subjective LSA wodr minimum (2nd)		
		*Subjective LSA minimum* (3rd)		

Features with SHAP values that are largely spread out across the x-axis, i.e., indicative of both directions, are not included in the table. Feature names in italic = features with significant differences between groups (Kruskal–Wallis test).

## 4 Discussion

The present study aimed at detecting relevant linguistic markers that could classify French-speaking UHR patients by their status at T0 and seeking to explain the classification results with regard to linguistic manifestations observed in the clinic. The results showed that our model based on XGBoost, SMOTE, and the SHAP values could get good performance through the interplay of the four linguistic markers obtained from a feature importance method using the SHAP values on the original data. These mean absolute SHAP values as feature importance revealed that the two uppermost features pertained to semantic coherence, the third most important to the use of “I,” and the last important feature was one of the disfluency-related elements, filled pauses. The two hypotheses thereby turned out to be true—semantic coherence and the use of “I” played a key role in the classification. The four linguistic markers identified pertain to weak coherence (intersubjective LSA minimum and subjective LSA wodr minimum, i.e., the lowest LSA score in each patient), self-related subject pronoun (the proportion of “I” to the total number of words), and disfluency (filled pauses).

Semantic incoherence has been reported to be a linguistic characteristic in FEP or schizophrenia (8, 10, 20, 40–42, 45, 46, 56). It is noteworthy that lower minimum scores contribute positively to FEP and negatively to NAR regardless of the LSA type. Higher minimum scores in subjective LSA appear to have a positive impact on classifying AR. The feature intersubjective LSA minimum turned out to have significant differences in Kruskal–Wallis and Dunn’s tests and a much greater impact on predictions than the other markers. This type of coherence was calculated between consecutive pairs of speech turns. Studies on coherence have been focused on patients’ utterances (40–44, 49, 52) like subjective LSA wodr (only-patient LSA) in our study. A dialogue is constructed within the framework of turn-taking described as a type of social organization that is implicated in speech exchange systems (103). For a dialogue to be coherent, a response should be fluent, consistent, context related (104), and the respondent should understand conventional meaning and catch their interlocutor’s intention. Dialogue coherence is thereby grounded in Speech Act Theory (105, 106) as well as related theories on conversation analysis and discursive pragmatics (107–109), wherein semantics and pragmatics are entailed. This weak dialogue coherence could partly explain some occasional strange speech and social interaction impairment in patients. Higher median values in subjective LSA contribute positively to NAR classification, whereas higher subjective LSA wodr median scores have a negative impact on FEP. Taken together, these results suggest that weak coherence is a marker of FEP even though it is still somewhat premature to generalize this finding due to the small sample size of FEP in the current study.

The use of the first-person singular pronouns in schizophrenia has been explored in some studies whose results were opposite to one another. When compared to patients with mood disorder, schizophrenics used fewer first-person singular pronouns (26) whereas these pronouns were more frequent in individuals with schizophrenia than healthy controls (28, 29, 56). The present study focused on the first-person singular subject pronoun “I.” The results showed no significant difference between groups, and higher and lower scores of “I” in FEP do not provide unequivocal contribution types contrary to what has been reported in the literature. However, more frequent use of “I” has a positive impact on AR classification, whereas it contributes negatively to NAR. The difference between the findings in the aforementioned studies and ours could be due to the differences in the populations compared (mood disorder vs. schizophrenia, healthy individuals vs. people with schizophrenia, NAR vs. FEP, and AR vs. FEP) and the pronouns compared (first-person singular pronouns; first-person singular subjective pronoun). The frequency of “I” in this study allowed differentiating between NAR and AR. The more frequent use of “I” in AR might indicate their more intense emotional distress compared to the NAR group as the statuses are the outcome of the CAARMS that assesses “emotional disturbance” in one of the seven subscales. Rude and colleagues showed that depressed college students used “I” more frequently—not the other first-person singular pronouns such as “me” or “myself”—than non-depressed peers (110). The differentiation between NAR and AR by the frequency of “I” might be indicative of more self-centered speech of AR and explained by their considering the self to be a solitary actor/agent as proposed by Rude and colleagues in (110). The meaning of higher and lower values in the frequency of “I” found in both directions in FEP is unclear and intriguing to us, but it might be partly explained by current affective disorders that turned out to be significantly more common in at-risk mental state than FEP (111). This claim does not refute the interpretation of the aforementioned differentiation between NAR and AR.

A filled pause is an uttered sound that fills a momentary interruption in speech production. When considered a pragmatic function, it has several functions such as discourse planning and structuring, and turn-taking (112) by signaling delays when a speaker stalls for time to retrieve information and wishes to continue their utterance (113). When considered a speech disfluency element, filled pauses are symptomatic of production difficulties (114). In the present study, the feature filled pauses is another marker that allows differentiation between NAR and AR. Its higher values contribute positively to NAR and negatively to AR. No impact of this disfluency element is observed on FEP classification. Another disfluency element, abandonment/auto-interruption, plays a role in classifying AR. When its scores are higher, it has a negative impact on AR predictions. It has been reported that patients with schizophrenia use fewer filled pauses (74, 115, 116) and produce longer filled pauses than healthy controls (117). Interestingly, Costa and Silva found that filled pauses before personal pronouns produced by patients with schizophrenia were twice as long as others, and the pronouns are mostly first-person singular pronouns (117). It was argued by the authors that their result could be explained by patients’ possible difficulties with self-reference. Filled pauses have ambivalent roles as mentioned above—they not only help speech production but also indicate hesitations and difficulties. Lower values in filled pauses in AR in this study, and fewer thereof in FEP in the literature, could be interpreted as indicative of somewhat disturbed pragmatic functions rather than speech disfluency. No contribution of filled pauses to FEP predictions contrary to what has been reported in the literature may be due to different populations compared (schizophrenia vs. FEP) and the small number of FEP patients in the current study.

The present exploratory study used recordings of the first consultations, a non-invasive method that does not transcend the classic healthcare frames, while allowing data collection under the same conditions for all participants. Our results provided evidence that a small number of linguistic markers without demographic or clinical data could classify UHR patients even at T0, that is, when patients do probably not present with obvious abnormalities in language behaviors. Besides, even healthy controls can experience mild language abnormalities (118), which could make language analyses more subtle and complicated. It should be pointed out that even though the AR patient at T0 who was misclassified into NAR is a single case of the kind in the present study, this misclassification—along with the other seven patients with their statuses at T2 who were correctly classified—is encouraging. It should cautiously be noted that the small number of FEP along with possible linguistic and cultural differences could make it somewhat delicate to generalize the results. However, the possible linguistic and cultural factor may not intervene in FTD as a systemic review article suggests a three-factor FTD structure with two prominent dimensions (disorganization and negative dimensions) is likely consistent and robust across languages (119). As a number of studies in the literature have also shown disturbed semantic coherence in FEP and schizophrenia, it could be argued that at least semantic disturbances are a universal linguistic manifestation of patients with psychosis regardless of languages and cultures. The SHAP values provided a local interpretation or the contribution of each feature to the classification. Even some features, such as the frequency of “I,” filled pauses, subjective LSA wodr minimum, wherein no significant group difference was observed, showed distinctive differences in the directions of the SHAP values and/or the magnitude. These differences would more likely reflect very subtle differences between patient groups recorded at a very early stage of care in psychiatry than an overfitting issue, since the model went through a cross-validation phase, although it was with a small k value. The SHAP explainability method could thereby allow getting deeper insight into the linguistic characteristics and speech patterns of each category of patients, which could lead to improving diagnostic methods.

## 5 Limitation

The current study lacks FEP patients and the 2-year statuses of most patients. In addition, our dataset is relatively small and imbalanced, which led us to carrying out an exploratory study to test the feasibility and potential of a gradient boosting model using only linguistic data. With new transformer-based models, such as BERT and SBERT, as well as word-embedding models, like GloVe, LSA is considered by some to be outdated, despite its advantages, mainly because LSA does not consider word order and context. This weakness might be critical to clinical data. It would therefore be interesting to use a new model combining LSA and BERT (BERT-LSA) (120) or other models in a future study. The inclusion of more patients and their statuses at T2 would allow more robust models and more accurate model performance evaluations. It is therefore planned to continue to record UHR patients, include more FEP, and analyze their speech using more classifiers for performance comparisons in search of a good diagnostic aid tool.

## Data Availability

The datasets presented in this article are not readily available due to medical confidentiality. Requests to access the datasets should be directed to D-HK-D, dh.kimdufor@gmail.com.
